# Wasted Alveolar Processes and Gums

**Published:** 1870-04

**Authors:** W. H. Atkinson


DENTAL REGISTER.

VOL. XXIV.] APRIL, 1870. [NO. 4.

### Original Communications.

### WASTED ALVEOLAR PROCESSES AND GUMS.

BY PROF. W. H. ATKINSON.

Read before the Southern Dental Association, April 14th, 1870.

To ask for remarks upon this particular subject, requires more of him who is to answer, than enters into the minds of most that ask it; for it involves more minute knowledge of anatomy and physiology than those claiming to be the best pathologists extant have yet attained. The claim of knowing, opens to him who makes it a multitude of responsibilities, legitimate and illegitimate, perceived and unperceived, of which he will be made aware so soon as he attempts to reply to the satisfaction of all who endeavor to perplex and annoy him, that they may show how sharp they are in propounding deep queries. The greatest difficulty in the way of clear elucidation and explanation to Dentists as a class, is their utter lack of knowledge in histology. Coming from the shop rather than from the college into the practice of Dentistry, is the principal cause of this lack. And even those who have availed themselves of all the colleges have taught, are yet in the verriest alphabet of histological science.

We speak of healthy and unhealthy systems and organs, and assume to be able to deduce their status from inspection of the exteriors, utterly forgetting, or ignoring that the elements of organs and systems must have departed very far from the healthy standard before it be possible to determine that fact by observation. In fact we must become familiar with the character of bodies too small to be seen by the unaided natural sight, before we can by possibility comprehend in any clear manner the subject of function.

Our first duty is to determine the conditions of the case when presented for our advice and treatment. Let us take a case of "wasted alveolar processes" as it presents. How shall we proceed? Why, look at and examine it, of course every one says at once, yes, that is very well so far as it goes, but the important query is, how far does it go? and what is the process of looking at and examining? I will not pretend to designate the process that, as a rule, passes for examination; but that it is too frequently a hasty, careless glance at the parts, and as hasty a decision, that it is probably an hereditary condition or taint that nothing now can prevent or remedy; is of too frequent occurrence to permit us to doubt it.

If this is what has passed and is passing for diagnosis and prognosis in these cases, is it not worth our while to ask what the process ought to be, what it really involves, and how we ought to proceed?

First, then, the process ought to be such as to enable us to definitely determine the exact state of the case; and second, it involves a knowledge of the formation, growth and nutrition of the parts concerned; and third, we ought to proceed on this basis of a knowledge of the primates which elaborate the function of the territory involved.

It is evident, to carry out this plan of procedure we must be able to comprehend the ever changing condition of the anatomical elements, by which the pabulum is appropriated, and the non-pabulum is rejected, because the first is needed, and the second is not required at the time. It is precisely this alternation of work and play, or labor and rest of these minute bodies that constitute normal function, accepting that of which they stand in need and rejecting that not required to maintain them in proper size and activity for the use of the system to which they belong, and which they in the aggregate constitute. In the light of this statement, it will be evident to all prepared to take it in, that there are but two primal conditions of interference with the equable performance of the all important work of maintaining these elements of the tissues in health. And these two basic elements of pathological activity are simply *deficiency* and *excess* of the work of normal function. If then, laziness and pulse activity lay at the very threshold of aberant function in the simplest elemental bodies of our systems, shall we regard it as a wonder that the same or like divergences from normality, find their unwelcome way into the tissues, organs, systems and minds which constitute us individuals, and lay the foundation of all our responsibilities, mental and material, moral and physical?

Let us now enquire, what are the elemental bodies of the alveolar processes? And answer: the alveolar process proper is that which we denominate bone. And, now, what are the elements of bone? And reply: not as the schools and erudite authorities teach, "an animal substance known by a specific form of cell," but as nature and common sense, honestly interpreted and exercised demonstrate, a mixed example of mineral and vegetable elements, produced and maintained in specific degree of differentiation by animal surroundings—these being first, Pabulum; second, Nervous; and third, Vascular in character. These animal bodies themselves partake, in their degree, of the character of vegetable and mineral modes of maintaining existence.

The vessels and nerves that creep in congeries and fasciculus, capillary and filament, over and among the osseous substance of the alveolar processes, come from and return to the general centers of heart and lung, brain and spinal cord, that the afflux and efflux of affete and effete pabulum may maintain the harmonious relations of health among these diversely endowed elemental bodies.

If the processes of the production and nutrition of the teeth and alveolar processes be comprehended, we are prepared to understand that the enamel is maintained in sound condition exactly as crystals are in the mineral domain of material bodies: and that the dentine is kept in condition by the to-and-fro movement of fluid in the passages that admit it, after the manner of the vegetable, and that bone is supplied by a mixed expression of these mineral and vegetable modes of endowment. Cement may be in this sense classified as bone, although it is the compromise of nutrient activity just midway between dentine and bone proper, of which the alveolar process is a legitimate example.

The process is surrounded by a fleshy body called gum, composed of tissues, wrought out of the blood corpuscles, and collagenous fibres, that is well supplied with bloodvessels, but sparingly with nerves, especially, the margins around the necks of the teeth where its connective tissue fibres are wrought into the "legamentum dentium."

We are now ready to assert that there are two forms of "wasting of the alveolar processes," namely, solution of the earthy material, in the membranous or collagenous matrix, the latter remaining entire and in place; and solution of both matrix and mineral salts. The latter occurs under two forms also, the first by atrophy of the elemental constituents of the margins of the processes, and the former by a loss denominated ulcerations or caries of the edges of the process.

Ulceration is resultant upon local expression of systemic debility or local uncleanness. Atrophy results from pauperized blood or mechanical interference. Mechanical disturbances have also two doors of entrance: first, accumulations of foreign matters of benign character, and second, injudicious efforts at cleansing the teeth with unsuitable instruments and dentifrices.

The production of the alveolar processes is part and parcel of that known as the process of dentition. And the only reason why the alveolar processes are liable to wasting and reproduction to a degree not known to the harder two of the dental tissues, namely, enamel and dentine, is in consequence of the difference in the mode of growth and nutrition by which they are produced and maintained as already hinted.

In all cases of wasting dependent upon constitutional degeneracy, general treatment combined with local cleanliness will be the remedy.

In such instances as are brought about by excessive and misdirected brushing, the remedy is to correct the manner and amount of effort.

And in cases of ulceration or caries of the alveolar processes, proceed to remove all foreign material, by carefully and scrupulously cleansing and polishing the necks of the teeth and margins of the processes, and then wetting the raw surfaces of process, tooth and gum with a strong solution of the chloride of zinc. After which establish correct hygienic habits, and all is well.

And now for correct hygiene of the mouth and we have done for the present. Oral hygiene, like general hygiene, is almost unknown and uncared for, other than for mere "appearance sake," hence little studied and most wretchedly practiced.

Here, as everywhere, the opposite of the truth has been taught and prevails? In one word, then, we ought to *always* brush the denture *in a line with the length of the teeth*, (and *never across* it as the *universal custom is*,) and *from* the gums toward, and over and *between* the crowns of the teeth, so as to harden the margins of the gums and cause them to close up tightly the line of junction with the necks of the teeth, and thus keep the teeth well polished on all sides. Any amount of use of brush and dentifrice necessary to accomplish this should be strenuously insisted upon, especially in the children and younger patients under our care.

In other words, polish off the "persistent capsule" or "Nasmyths membrane" as it has been called, so soon as the crowns of the temporary or permanent teeth emerge from the gums, by any means that will secure a fine polish to the distal ends of the enamel rods; then keep up the polish by the use of brush and dentifrice as already stated during life.

To be quite clear, let me once more say: brush the *upper* teeth *downward*, and the *lower* teeth *upward*, scrupulously avoiding any *in* and *out* motion of the brush, as it is this *pernicious practice* that so universally exposes the necks of the teeth on the outer side, in the mouths of all, so called, "good brushers."

So soon as these principles and practices shall prevail there will be no more cases of "wasted processes and gums" to perplex our minds and scourge our affections.

